# Analyzing NBA player positions and interactions with density-functional fluctuation theory

**DOI:** 10.1038/s41598-025-04953-x

**Published:** 2025-06-05

**Authors:** Boris Barron, Nathan Sitaraman, Tomás Arias

**Affiliations:** 1 Department of Physics, Cornell University, Ithaca, 14850 United States; 2 Max Planck Institute for Demographic Research, Rostock, 18057 Germany

**Keywords:** Biological physics, Statistical physics

## Abstract

The increasing availability of high-precision player-tracking data in sports—centimeter-precision positional information of athletes captured dozens of times per second—has the potential to improve the quantification of player abilities and overall team strategies. Working toward achieving this quantification, we adapt density-functional fluctuation theory (DFFT) to infer spatial preferences and player-to-player interactions in National Basketball Association (NBA) basketball. We first demonstrate several foundational results, including the ability of DFFT to predict the location of a player to within 3% of the half-court area roughly half the time, and to provide a team-position-based metric that correlates strongly with play outcomes. Building on these results, we demonstrate that it is possible to improve player positioning and identify player-specific tendencies, such as the consistency with which a player positions himself to *help his team collectively* defend against 2-point or 3-point shots. Finally, we quantify how particular players attract the opposing team, with and without the ball, constituting the first advanced quantification of ‘player gravity’ that explicitly deconfounds the influence of teammate positioning.

## Introduction

High-resolution player tracking has been available in professional sports for just over a decade^1,2^. Although the potential implications of tracking data have been widely noted^1,3,4^, and a variety of machine learning^2,5,6^ as well as statistical techniques^7–10^ have been applied, utilizing spatio-temporal information in sports is an emerging field^4^. The overarching challenge is to develop a high-quality analysis that utilizes tracking data to understand team strategy, player ability, and play outcomes^1^. In this work, we provide a potential solution to this challenge by adapting a physics-based approach that is intuitive and has substantial predictive power—an approach that can evaluate not only nuanced player tendencies but also, for example, predict how play-outcome probabilities change if the location of a player is altered at a crucial moment.

Although relatively new in the context of sports analytics, the task of inferring fundamental relationships from positional data is a recurring challenge in both the physical^11,12^ and social^13,14^ sciences. One particularly notable framework for analyzing positional data in collective systems is density-functional theory (DFT)^15^, a Nobel Prize–winning technique that reduces complex many-body positional information to a more tractable form based on spatial densities. Originally introduced to describe the electronic ground states of quantum mechanical systems of electrons and nuclei^16,17^, DFT has since been extended to various regimes: time-dependent DFT (TD-DFT)^18^ for time-varying behavior, and current-DFT for systems with magnetic interactions^19^. Classical (non-quantum) versions of DFT have been developed^20–22^ and have found wide application in soft condensed-matter physics^23,24^, including time-dependent formulations such as dynamical DFT (DDFT)^25^. More recently, classical DFT has been extended to settings where the underlying interactions are unknown—a formulation known as density-functional fluctuation theory (DFFT)^26^. DFFT seeks to infer interactions (*e.g.*, between players in sports) and spatial preferences directly from fluctuations in positional data. Despite its recent introduction, DFFT has already been applied to systems as diverse as insect group organization^26^, racial segregation in cities^27,28^, and simulations of crowd dynamics in public spaces^29^.

To understand the capability of DFFT, first consider what could be inferred from player-tracking data if there were no data limitations (every possible position is present in the dataset a large number of times). In this limit, the inference problem is statistical: *i.e.,* given a position—the locations of the ball and all of the players—it is straightforward to determine the probability that the position results in 0, 2, or 3 points for the offense from prior instances of this exact position in the dataset. It would also be straightforward to determine how the probability of a particular outcome would have changed if a player were present at a different location on the court, if one player had been exchanged for another, or if any other aspect of team positioning had been altered. The benefit of DFFT is that it allows the determination of these probabilities using datasets that are of a realistic size while employing only a minimal set of assumptions.

In particular, the salient assumption in DFFT is that precise player positioning can be transformed into ‘player densities’ which provide an aggregate understanding of a position (*e.g.,* there are no players close to the hoop and a substantial defensive presence near the 3-point line). First, we describe how this transformation can be performed and how outcome probabilities can be determined. The second step in DFFT is parameter reduction, where we demonstrate that the number of parameters necessary to describe these probabilities can be reduced by two orders of magnitude while retaining similar levels of efficacy. In particular, this allows DFFT to work with small datasets that arise, for example, from a specific player being present on the court. Overall, this DFFT parametrization can be interpreted as quantifying location-based preferences and, separately, player-to-player interactions, which together are able to capture subtle aspects of the game.

After demonstrating the efficacy of DFFT for predicting player locations and play outcomes, we then build on this capability to determine which specific defensive players in the National Basketball Association (NBA) are consistently in strong defensive positions. We emphasize that this quantification does not simply capture how often the player is directly defending the ball, nor does it consider how physical attributes like height and leaping ability may contribute to a successful defense. Rather, it identifies if, based on the locations of the other players and the ball, the player is *well-positioned to help his team defend* against 2-point or 3-point outcomes. We then consider the offense, where we quantify how strongly specific players attract the defense—*i.e*., ‘player gravity’ ^30^. While prior work has estimated gravity based on defensive player attention^31^, or overall spacing between the defense^32^, our DFFT-based measure has the natural interpretation of gravity as ‘affecting defensive density’ and is the first—to our knowledge—that explicitly deconfounds the influence of teammate positioning. Finally, we describe several exciting directions for future work, including how offensive players affect the defense at a distance, how quickly particular defensive players anticipate play outcomes—colloquially known as ‘defensive IQ’^33,34^—and how DFFT can be used to optimize player positioning.

## Methodology

### Player densities

The first step is to transform tracking data into a form that allows us to quantify ‘similarity’ between positions. Such a transformation extends traditional sport metrics that explicitly *presume* which positions are ‘similar’. For example, 3-point field-goal percentage (3P%) provides a description of player skill that reduces data from all observed situations (opponent positioning, defense composition, *etc.*) to simply the percentage of successful shots from beyond the 3-point line^35,36^. In this metric, all 3-point shot attempts by a player are viewed as similar positions that collectively quantify 3P%. A straightforward incorporation of tracking data allows determination of a player’s 3-point percentage in situations where the nearest defender is at a specified distance^37^ or using this distance as one aspect for estimating shot quality^2,31^. Using DFFT, we aim to provide a general definition of similar positions with predictive power under a broad class of positional changes.

**Fig. 1:**
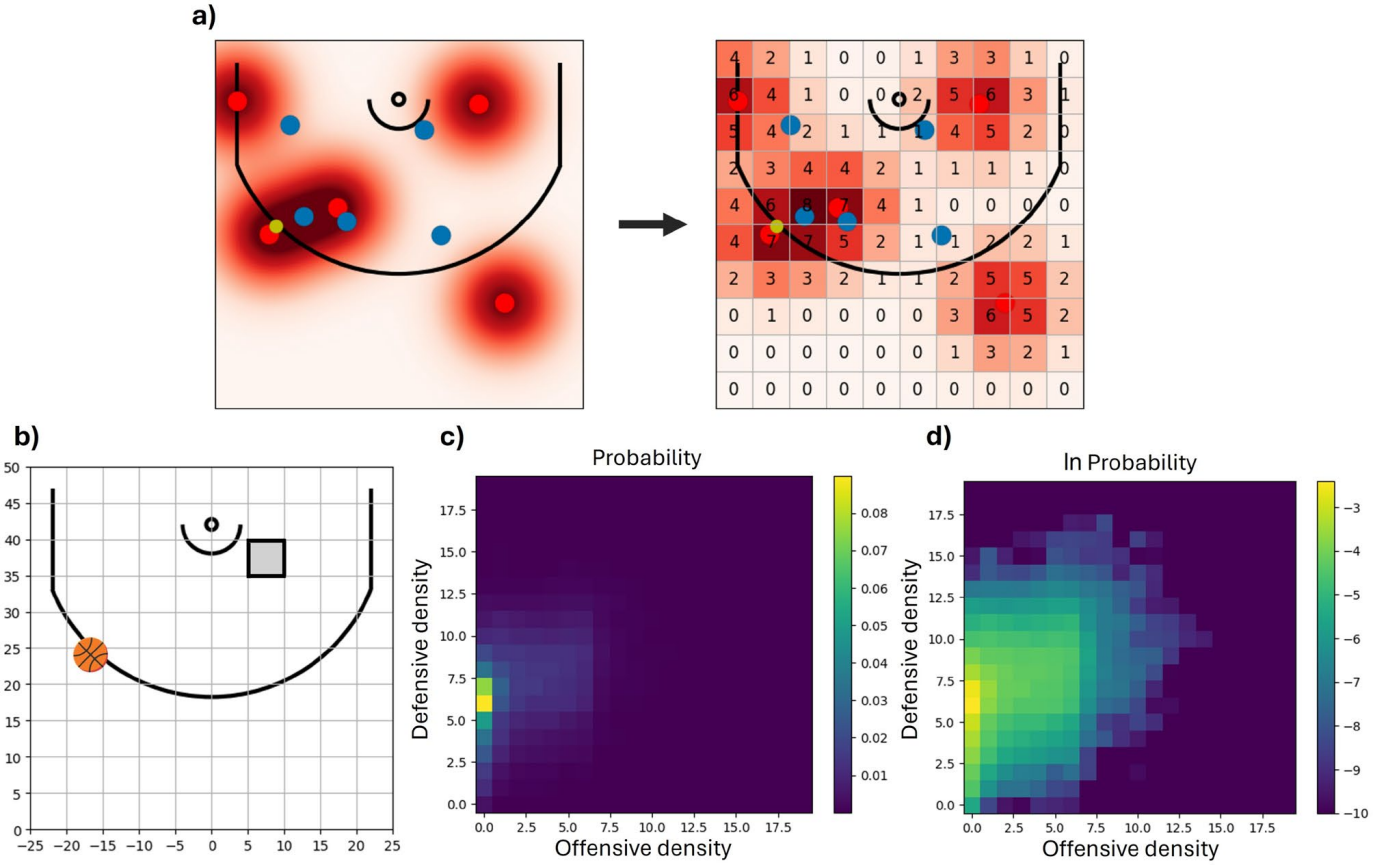
Density formulation of player locations. (**a**) Offensive player locations (red circles) of a position are transformed into a matrix of offensive densities taking discrete values throughout the half-court. (Defensive players, the blue circles, are transformed in the same way to obtain defensive densities.) (**b**) The overall dataset can be filtered using a set of conditions, such as the ball (basketball icon) being near location [-16, 24] and the offense attempting a shot within the next 3 seconds that the defense successfully prevents. (**c**) Resulting joint probability distribution of defensive (vertical axis) and offensive (horizontal axis) density values (colormap) in the grey bin from (**b**) for the considered conditions. (**d**) Colormap of natural logarithm of the probability distribution in (**c**). The associated joint probability distribution in the grey bin is categorized by high defensive and low offensive densities, with a visible positive correlation (more evident in (**d**)) between the offensive and defensive densities.

The basis of density-functional theory (DFT) is the representation of a many-body system in terms of interacting densities ^16^. The salient assumption in density-functional fluctuation theory (DFFT) is that a system of interacting agents (fruit flies, people) can be similarly formulated despite the underlying interactions being unknown. When the number of interacting agents is large, these densities are constructed by counting how many agents are localized in a region of a reasonable size. However, as the number of interacting agents in basketball is small (there are only 10 players), we instead consider each player as generating a ‘player density’ with a tapering spatial extent. In this work, the density generated by a player will be represented by a two-dimensional (2D) Gaussian with a standard deviation of 5 feet, $\sigma =5$ ft, centered at the player’s actual location, and the density for the corresponding team will be the sum of the Gaussians of the associated players. The left side of Fig. 1a presents an example of the resulting density for the offensive players of a position. Note that this definition treats players of the same team as being indistinguishable—if two offensive players on the team are swapped the resulting density is unchanged—although, as we demonstrate in our results section, this still allows us to draw player-specific conclusions.

The reason for introducing a player density is not to directly quantify the spatial influence of each player—we are not claiming that the influence of a player is a Gaussian of 5 feet—but rather to be able to identify similar positions with minimal assumptions. For example, when a player is moved by only $\approx$ 1 foot in any direction, this causes a slight change in densities throughout the half-court, allowing us to quantify the similarity between the original and altered position and identify these positions as being nearly identical. Through the use of densities, consideration of player locations is replaced by an aggregate understanding of a position; *e.g.*, “there is a lot of offensive density near the basket with a small defensive density.” This approach is intended to retain the important aspects of the game while allowing us to consider probability distributions for the densities under various scenarios. In this work, densities will be evaluated (representing the average density over an area) at 100 locations throughout the half-court, a 10$\times$10 grid of bins ($N_{bins}=100$), each being 5$\times$5 feet, as shown on the right in Fig. 1a.

Additionally, as the models arising in DFFT are generally non-parametric, it is beneficial to discretize the allowable density levels in each bin. In this work, we set the discretization to 20 levels ($N_{densities}=20$); with 0 corresponding to the absence of density, 7 corresponding to a player in the center of the bin far from his teammates, and 19 corresponding to three defensive (or offensive) players standing close to each other near the center of the bin. Roughly, this density discretization can be thought of as setting a spatial resolution of the players to around half a foot: moving a player by less than half a foot typically leaves density values unchanged. The right side of Fig. 1a provides an example of the discretized densities for the offense.

### Probabilistic model for player densities

In order to quantitatively evaluate a position—the locations of the players and the ball at a single moment of a play—it is necessary to determine the relationship between densities and play outcome (*e.g.,* scoring) probabilities. First, note that, after the dataset is filtered using a set of conditions (*e.g.*, ball location, time on clock, scoring result), it is straightforward to evaluate the joint probability that a location on the half-court has simultaneously an offensive density, $n_o$, and a defensive density, $n_d$. In particular, with our density discretization to 20 density levels, the density distribution represents the probability that a location (bin) of the half-court takes one of $N_{densities}^2 = 400$ states (combination of offensive and defensive densities). Direct representation of the distributions in all of the 100 bins then requires $N_{bins}N_{densities}^2= 40,000$ parameters.

As a specific example, Fig. 1b-1d demonstrate the density distribution for a bin near the hoop (bin centered at [x = 7.5 feet, y = 37.5 feet]) obtained from positions in which a shot is taken within 3 seconds, the defense is successful ($R_{shot} = 0$, meaning 0 points for the offense at the conclusion of the play), and the ball is located near [-16, 24]. Under these conditions, the density distribution in this bin (Fig. 1c) has an average defensive density of just under 7, equivalent to a defensive player standing at the center of this bin—being in a strong position to get a weak-side rebound. Meanwhile, the average offensive density under these conditions is much smaller, just over 2, which roughly represents the average offensive density expected across all bins. Furthermore, $\ln P_b(n_o, n_d)$, shown in Fig. 1d, clearly shows a positive correlation between offensive and defensive densities: high offensive densities in this bin are almost always accompanied by high defensive densities. Thus, our density formulation provides direct quantitative evidence of the obvious fact that offensive and defensive players are interacting.

The utility of density distributions comes primarily from their relationship with play outcome likelihoods. Given a position, the loglikelihood can be quantified

1 $$\begin{aligned} \ln \tilde{P} = \sum _b \ln \left( P_b (n_{o,b}, n_{d,b}) \right) , \end{aligned}$$

where $n_{o,b}$ and $n_{d,b}$ are the offensive and defensive densities of the position in bin *b*, and $P_b(n_o, n_d)$ is the density distribution for bin *b* that is obtained from all positions in the dataset that satisfy a given set of conditions (*e.g.,* play outcome). The greater the loglikelihood the more likely are those conditions, and thus evaluating loglikelihoods for different outcomes provides information on their relative likelihood. Finally, we note that, because we expect the densities in nearby bins to be correlated, the loglikelihood is not exactly the logarithm of the overall probability—hence we represent it as $\ln \tilde{P}$ rather than $\ln P$—although our results demonstrate empirically that the relationship between $\ln \tilde{P}$ and outcome probabilities is straightforward.

### Modeling player densities with DFFT

To determine the density probability distributions, $P_b(n_o, n_d)$, we need sufficient data satisfying the desired conditions. If the conditions are likely to occur (*e.g.,* the result of the play was a missed shot), it may be viable to estimate all of the necessary 40, 000 parameters to determine the density probability distributions, which we will refer to as a “direct probabilistic” (DP) model for the densities. However, reducing the number of parameters is useful for two reasons: (1) it reduces overfitting, allowing for more consistent performance between the training and testing datasets, and (2) it allows for the use of smaller datasets and therefore the application of rather specific conditions, such as a specific player being present on the half-court.

To accomplish this reduction in parameters, we directly employ concepts from density-functional fluctuation theory (DFFT). The underlying idea is that it is unnecessary to require 400 parameters per each of the 100 bins because there are aspects of the density distributions that should be universal across bins. Then to represent the density distributions for each of the bins, bin-specific parameters are introduced which capture the most salient distribution-altering properties among the bins, with a common DFFT choice being the average density values. This choice is particularly reasonable for basketball because the average offensive and defensive densities vary substantially throughout the half-court, and these average-density parameters can be interpreted as identifying a location-based preference for the offensive and defensive players.

Following the DFFT work of Valderrama et al.^26^, Chen et al.^27^, and Barron et al.^38^, the joint density distribution for bin *b* in the simplest case is given by

2 $$\begin{aligned} \tilde{P}_{b}(n_o, n_d) = \frac{1}{Z}e^{-n_o v_{o,b} - n_d v_{d,b}-f(n_o, n_d)}, \end{aligned}$$

where the values for the parameters are obtained by

3 $$\begin{aligned} \{f(n_o, n_d),v_{o,b}, v_{d,b}\}= \underset{f(n_o, n_d)}{\text {argmin}} \sum _b \underset{v_{o,b}, v_{d,b}}{\text {argmin}} \sum _{n_o, n_d} P_b(n_o, n_d) \ln \left( \frac{P_{b}(n_o, n_d)}{\tilde{P}_b(n_o, n_d)} \right) , \end{aligned}$$

where $P_{b}(n_o, n_d)$ is the actual distribution in bin *b* and $\tilde{P}_{b}(n_o, n_d)$ is the corresponding DFFT approximation for this distribution. (See Supplemental Section S1 for derivation of this DFFT model.). Conceptually, $f(n_o, n_d)$ encompasses the best estimate for a bin-universal distribution—capturing interactions among players—based on the distributions observed across all bins, while $v_{o,b}$ and $v_{d,b}$ improve the estimate for the distribution in bin *b* by capturing bin-specific average densities, with lower values of $v_{o/d,b}$ representing bins *b* which are relatively more “attractive” or likely locations for the offense/defense, respectively. We note that the substantial success of the DFFT approach is due to the information-theoretic underpinning of the model construction process, which identifies the *form* of the model (*e.g.,* Eq. 2) that takes as an input precisely the desired quantities—in this case a bin-universal function and bin-specific average densities—to represent probability distributions. The end result is a reduction in the number of parameters from $N_{bins} N^2 _{densities} = 40,000$ down to $2 N_{bins}~+~N_{densities}^2 = 600$.

## Data

Our analysis in this paper uses Second Spectrum player tracking data from the beginning of the 2022-23 NBA season through January 20, 2023. We limited consideration to positions no more than 3 seconds prior to a shot attempt. We further exclude positions deemed to be ‘in transition’ between which team is in the offensive role: specifically, our criteria are that the average player position is on the offensive side of the court and at least 20 feet beyond the half-court line and that the average player speed along the long axis of the court is no more than 5 feet per second. For player-specific results, we considered the 50 players that were on the court most frequently in the dataset as well as Stephen Curry, because Curry is widely considered important for discussion of player gravity^30,31,39,40^. We take ball location into account by weighting positions in the dataset by distance from a target ball location using a Gaussian with $\sigma = 2$ ft. The locations we considered for the ball are approximately uniform throughout the half-court and include 36 locations inside the 3-point line and 53 locations outside the 3-point line. Finally, our ‘testing set’ positions were limited to positions where the ball was within 2 feet of one of these 89 locations. (See Supplemental Section S2 for details on how ball location was considered.)

## Efficacy

If DFFT is successful, then we should be able to compute anything using the DFFT model (600 parameters) which could be obtained from a direct probabilistic (DP) model of the density distributions (40,000 parameters), with the statistical advantages that arise from fewer parameters. This means that by ‘training’ the DFFT model on subsets of the data satisfying specific conditions (teams, ball location, outcome,* etc.*), we can (1) predict where a player is likely to be based on the locations of the other players and (2) evaluate the probabilities of various outcomes for a position. In particular, performing these tests is foundational to establish the efficacy of DFFT in a basketball setting, and the resulting performance provides confidence in applying DFFT to datasets that are too small for consideration using the direct probabilistic approach.

### Locating a player

For the first efficacy test, suppose that we know the location of 9 players (5 offensive + 4 defensive) as well as the ball, and we are trying to determine the location of the last defensive player. We can consider placing this ‘hidden’ player in a variety of locations (such as a grid throughout the half-court with a spacing of half a foot, the resolution of our density discretization) and then use Eq. (1) to evaluate the loglikelihood ln$\tilde{P}$ of the overall position given each hypothetical location for the player.

**Fig. 2:**
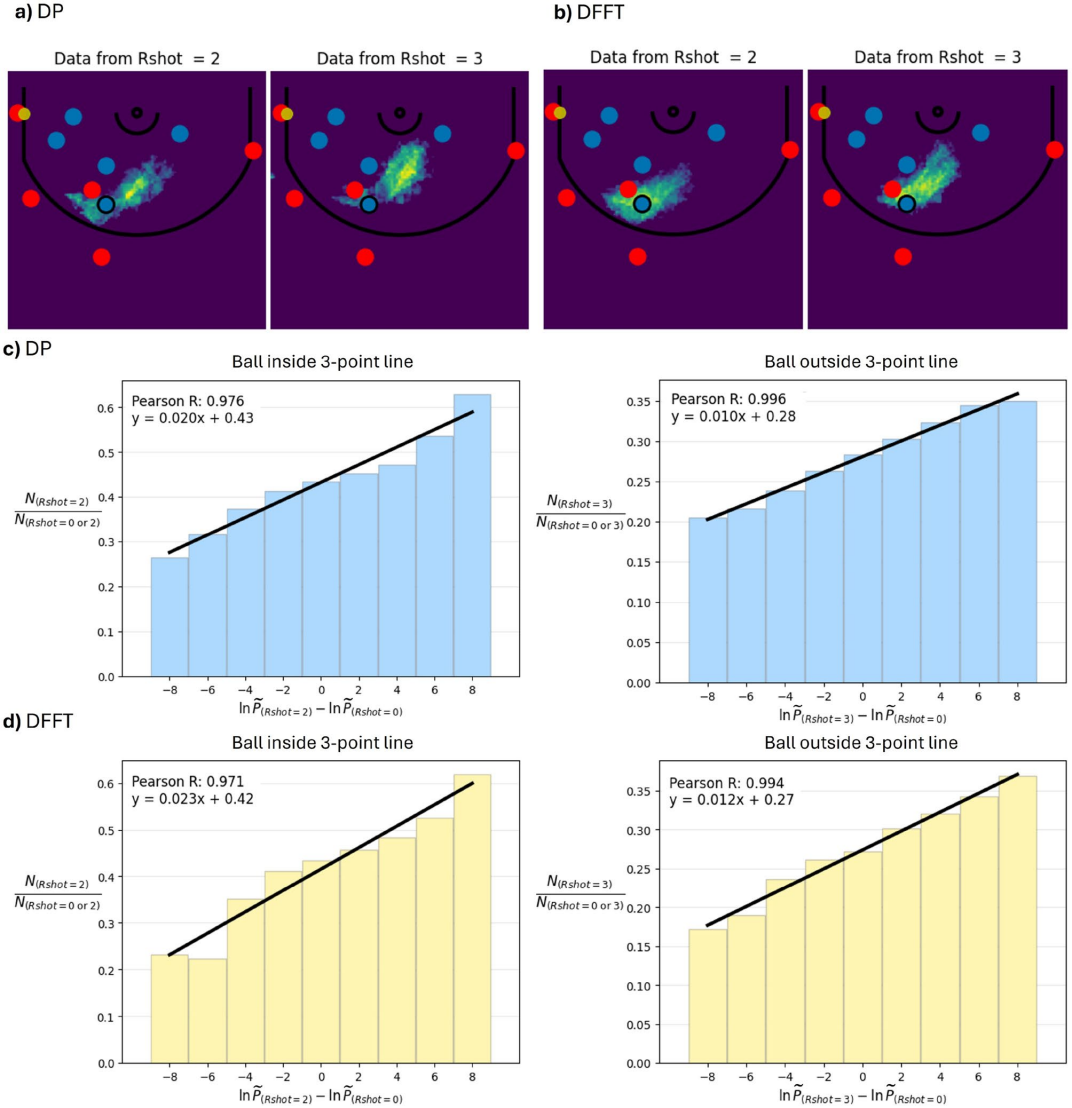
Efficacy of DP and DFFT approaches in predicting player locations and play outcomes. (**a**) and (**b**) Predicted distributions (colormap) for the location of a ‘hidden player’ (blue circle with black outline) based on the locations of the other 9 players (defense/offense in blue/red) and the ball (yellow) using (**a**) direct probabilistic (DP) and (**b**) density-functional fluctuation theory (DFFT) approaches from positions corresponding to $R_{shot} = 2$ or 3 point outcomes for the offense. Colormap shows the most likely positions for 5% of the area of the half-court, with yellow shades indicating more probable locations. This procedure locates the hidden player to within such an area (5% of the half-court) 60-70% of the time, depending on the dataset. (**c**) and (**d**) Predicting shot outcomes using ln$\tilde{P}$ differences: $R_{shot} = 0$ vs $R_{shot} = 2$ outcome (left column), and $R_{shot} = 0$ vs $R_{shot} = 3$ outcome (right column), using (**c**) DP and (**d**) DFFT approaches. The correlations are evaluated for the ball being located *inside* the 3-point line for 2-point outcomes (left column) and *outside* the 3-point line for 3-point outcomes (right column). For example, when the ball is inside the 3-point line, positions that result in either $R_{shot} = 0$ or $R_{shot} = 2$ and are evaluated as having a ln$\tilde{P}_{Rshot = 2}$ - ln$\tilde{P}_{Rshot = 0} = 8$ have a 60% chance of resulting in $R_{shot} = 2$. This stands in contrast to positions evaluated as having a ln$\tilde{P}$ difference of -8, where less than 30% of these positions result in $R_{shot} = 2$. Note that these results are obtained for the testing set positions, consisting of plays that were not used for model training.

Figure 2a and 2b show the most probable locations for a hidden player in a position using datasets conditioned on the scoring outcome ($R_{shot}$ = 2 or 3). Notably, despite the reduction in the parameters afforded by DFFT, there is not only substantial agreement for the expected location of the player using both DP and DFFT approaches, but there is also agreement regarding subtle differences between 2- and 3-point outcomes, finding that $R_{shot}$ = 3 becomes more probable had the player been closer to the hoop. In fact, after training on 80% of the data, our DFFT approach generally identifies the defensive player location in the testing set (20% of data) to within 1% of the half-court area around a quarter of the time, within 3% around half of the time, and to within 5% nearly two-thirds of the time, with DP only marginally outperforming DFFT. (See Supplemental Figure S2 for details using datasets corresponding to $R_{shot} = 0, 2,3$.) Finally, due to the far fewer parameters involved, the DFFT approach is much less susceptible to overfitting and various artifacts. (See Supplemental Figure S3 for an example of this effect.)

### Position evaluation

Figure 2a and 2b suggest that there is general agreement on the probable locations of a player using datasets of different scoring outcomes. However, as noted above, there are important differences. The second efficacy test is to determine to what extent DFFT is able to capitalize on these subtle differences to identify scoring probabilities.

To quantify scoring probabilities for a position, we compute ln$\tilde{P}$ for a position using datasets where the defense was successful ($R_{shot}$ = 0) and, separately, datasets where the offense was successful (either $R_{shot}$ = 2 or 3). We can then use differences between these loglikelihoods to quantify the relative scoring probabilities for a position. Figure 2c and 2d demonstrate that ln$\tilde{P}$ differences indeed correlate strongly with scoring probabilities and in a highly linear fashion. In particular, considering positions of the testing set (plays not used for training) where the outcome was $R_{shot} = 0$ or 2 and the ball was inside the 3-point line, we find that for positions evaluated with DFFT as $\ln \tilde{P}_{Rshot = 2} - \ln \tilde{P}_{Rshot = 0} = 8$, more than 60% of these positions result in a 2-point outcome. In contrast, when $\ln \tilde{P}_{Rshot = 2} - \ln \tilde{P}_{Rshot = 0} = -8$, just over 20% of these positions result in a 2-point outcome. The slope, indicating percentage change per ln$\tilde{P}$ difference, was actually marginally greater with DFFT than DP, $2.3\%\big /\Delta \ln \tilde{P}$ compared to $2.0\%\big /\Delta \ln \tilde{P}$, respectively, meaning that loglikelihood differences using DFFT are actually slightly more informative than DP. Similarly, when the ball is outside the 3-point line, and we are comparing likelihoods of $R_{shot} = 0$ with $R_{shot} = 3$, we find $1.2\%\big /\Delta \ln \tilde{P}$ with DFFT compared to $1.0\%\big /\Delta \ln \tilde{P}$ with DP. In all of these cases, the Pearson correlation coefficient is above 0.97. (Supplemental Section S5 further demonstrates that strong correlations are maintained when the ball is at a fixed location and, separately, in scenarios where the ball location changes prior to a shot attempt.)

## Results

### Ranking defensive positioning

Building upon our results from the previous sections, we consider which defensive players consistently position themselves to prevent either 2-point or 3-point outcomes with DFFT using a rather nuanced approach. Our procedure begins by considering where a ‘typical’ defensive player is likely to be located in a position given the locations of the other players and the ball; specifically, we consider the most probable 10% of the area of the half-court for a typical player (following Section ‘Locating a Player’). We then evaluate the ln$\tilde{P}$ over this entire 10% region for three separate models: a model trained on successful-defense outcomes ($R_{shot}$ = 0), successful-offense $R_{shot}$ = 2, and successful-offense $R_{shot}$ = 3. For each specific player under consideration, we calculate the ln$\tilde{P}$ difference (for example, between $R_{shot}$ = 0 and $R_{shot}$ = 2) at his actual location and further subtract off the corresponding ln$\tilde{P}$ difference averaged over the entire 10% region. Doing this separately for the successful-offense models ($R_{shot}$ = 2 or 3) allows us to estimate how a player improves his team’s defense against either two- or three- pointers as compared to other reasonable locations the player could have taken. We refer to the average of this player-specific quantity (averaged over many positions for a particular player) as ‘ln$\tilde{P}$ defensive gain’ for two- and three- pointers. We note that we only consider positions where the player under consideration actually stands in one of the 10% most probable locations (determined using $R_{shot}$ = 0 data), which filters atypical plays from our evaluation while maintaining approximately 80% of defensive plays.

**Fig. 3:**
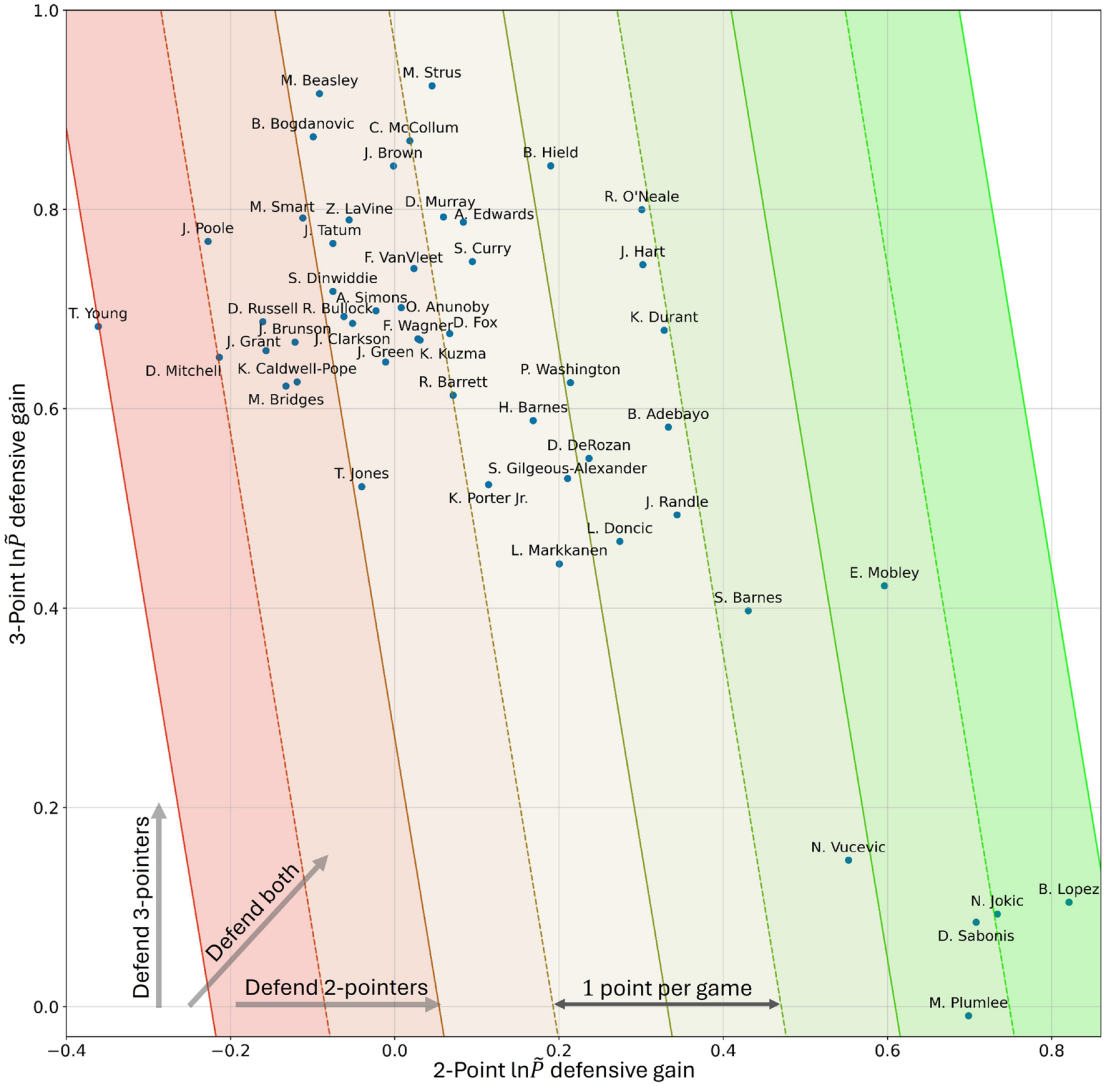
Specific players’ ln$\tilde{P}$ defensive gains for both 3-point (vertical axis) and 2-point (horizontal axis) shots evaluated at the time of the shot. The gains are evaluated from the specific player’s actual play locations as compared to defensive contributions of a typical NBA player (likely locations based on model trained on all players), with greater horizontal/vertical values indicating the extent to which particular players improve defensive positioning for preventing 2-point/3-point shots. Contour lines indicate approximate points saved per game based on 30 attempted 3-point shots and 60 attempted 2-point shots. The general anticorrelation reflects the tension between defending against 2- and 3-point shots.

As an example, if the average of the 10% area was $\ln \tilde{P}_{Rshot = 0} - \ln \tilde{P}_{Rshot = 2}$ = 4 over many positions, then the typical player is expected to help defend against 2-point shots to this extent based on the locations of the other players and the ball. However, when the difference is evaluated at the actual locations of the considered player the average may be $\ln \tilde{P}_{Rshot = 0} - \ln \tilde{P}_{Rshot = 2}<4$, indicating weaker 2-point defense, or $\ln \tilde{P}_{Rshot = 0} - \ln \tilde{P}_{Rshot = 2}$>4, indicating stronger 2-point defense. Figure 3 demonstrates the resulting defensive gains of each player, with the 2-point ln$\tilde{P}$ defensive gain along the horizontal axis (considering positions at the time of the shot with outcome $R_{shot} = 2$) and the 3-point ln$\tilde{P}$ defensive gain along the vertical axis (considering positions at the time of the shot with outcome $R_{shot} = 3$). (Note that we are only considering positions when the offense was successful so we can be certain that the player *should* have been trying to help defend against the respective 2-point or 3-point outcome: when the defense is successful, it is not entirely clear which outcome the player should have been defending against.)

Figure 3 shows the results from positions at the moment of a shot attempt, with 2-point attempts along the x-axis and 3-point attempts along the y-axis. As 2-point and 3-point shots attempts are considered separately, the best defenders should adjust their positioning to maximize their ln$\tilde{P}$ defensive gains along both axes. The results demonstrate that B. Lopez is the strongest 2-point defender at the time of a 2-point shot, with a ln$\tilde{P}$ defensive gain of more than 0.8, and T. Young is the weakest 2-point defender with a defensive “gain” of less than -0.35. Considering the vertical axis, M. Strus is the strongest 3-point defender at the time of the shot, with a ln$\tilde{P}$ gain of over 0.9, and M. Plumlee is the weakest, with a value near 0. It is notable that our approach clearly identifies players who primarily play the center position—*e.g.,* N. Vucevic, N. Jokic, B. Lopez, D. Sabonis, M. Plumlee^41^—as relatively strong 2-point defenders and relatively poor 3-point defenders. Meanwhile, players such as B. Hield, R. O’Neale, J. Hart, K. Durant, B. Adebayo, J. Randle, and E. Mobley defend both 2-point and 3-point shots to varying degrees, demonstrating their ability to appropriately adapt their location to assist their team’s defense.

These results can be used to provide a rough conversion to ‘points saved per game’ as a result of the positioning ability of various players. The oblique lines of Fig. 3 demonstrate how many points each player would save if they were present for 30 attempted 3-point shots and 60 attempted 2-point shots—roughly the shots attempted per game—using our DFFT correlation with scoring outcome at the time of shot. (See Supplemental Figure S6 for this correlation.) This analysis indicates that the difference between the best and worst defensive players corresponds to roughly 3 points saved per game, directly due to differences in defensive positioning. This analysis further finds that 2-point defensive specialists save more points than 3-point defensive specialists, which can be attributed to the higher attempt rate for 2-point shots and the stronger correlation between positioning and 2-point scoring outcomes. Improvements to defensive positioning, especially at the time of the shot, greatly reduce 2-point scoring probability. Improvements in 3-point defensive positioning, in contrast, may serve to discourage 3-point shot attempts (not directly captured in this analysis), rather than substantially reducing the 3-point shooting percentage.

### Player gravity

Determining how a particular offensive player attracts defensive players—player gravity—is complicated by gravity being location-dependent and potential confounding effects due to the locations of other offensive players. To account for these challenges, we train a DFFT model on positions that have a particular player (*e.g.,* L. Doncic) present at a specific location, and compare it to a DFFT model conditioned on *any* offensive player being present at the same location. We then consider an actual position for the offense (5 players) from the dataset—a position that has an offensive player at the considered location—and compute the average defensive densities that arise in the surrounding bins using both the player-specific and the typical-player models. For example, if the defensive density is expected to be higher in the surrounding bins with the DFFT model trained on L. Doncic than that of the typical player, then we can be confident that the increase in defensive density is due to the specific presence of Doncic. By applying both models to the same offensive position(s), the difference in defensive density between the models accounts for the confounding effect of the locations of the other offensive players. Finally, because the player-specific subsets of the data can become rather small, we note that we must use DFFT rather than DP for this application.

Our approach for determining player gravity has the direct advantage of identifying location dependencies. In particular, we evaluate each player’s gravity at eight different locations across the half-court. (In the actual computation, we consider all plays where the player under consideration is standing to within 5 feet of each target location.) The ‘local’ defensive density was then computed in the bin of the offensive player and the surrounding bins (*i.e.*, a nine-bin average, equivalent to a 15 by 15 foot square surrounding the player) for each of the eight considered half-court locations. To provide an intuitive scale for this ‘local’ gravity (change in local defensive density), we will use what we refer to as ‘ball equivalents’. We define *ball equivalents* using a linear map defined separately for each of the eight locations so that 0.00 corresponds to the “off-ball” local defensive density of a typical player (defined as the ball being more than 3 feet from the player’s location) and 1.00 corresponds to the “on-ball” local defensive density of a typical player (defined as the ball being within 3 feet).

**Fig. 4:**
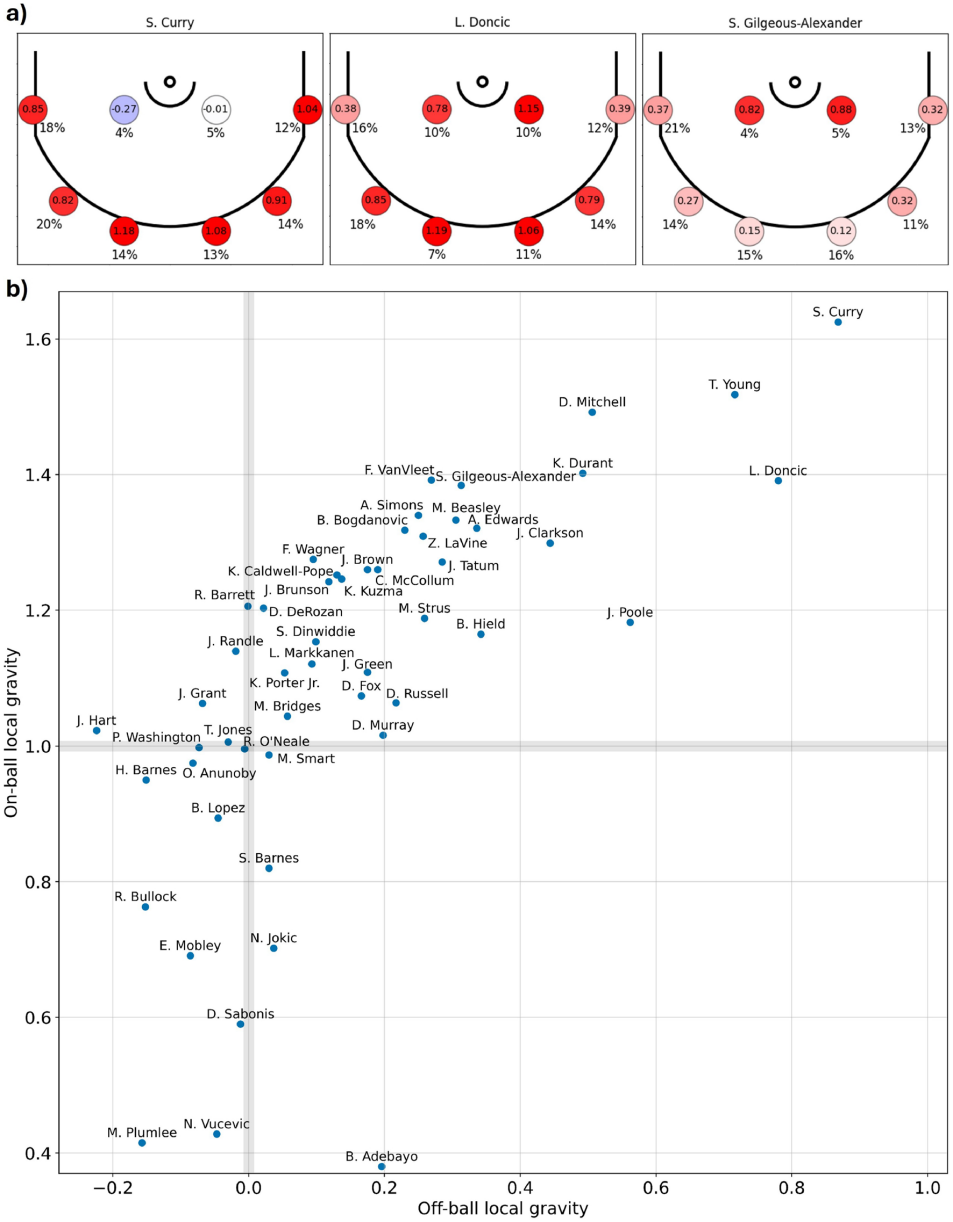
Advanced quantification of player local gravity. (**a**) Off-ball local gravity at various locations on the court for specific players, circles indicate locations with gravity represented by encircled value and shade. 0 corresponds to the average off-ball gravity of a typical player and 1 corresponds to the average on-ball gravity of a typical player at each of the eight locations. The percentile near each circle shows the fraction of the time the player spends at each location. (**b**) Average local gravity for players (labeled points) for on-ball (vertical axis) and off-ball (horizontal axis) cases weighted by the fraction of time that the specific player spends at each location. As these are the most frequently occurring players in the dataset, they appropriately tend to have higher-than-average gravity.

Figure 4a shows location specific off-ball local gravity of S. Curry, L. Doncic, and S. Gilgeous-Alexander. We find that S. Curry, a 3-point specialist, has very high gravity near the 3-point line—his off-ball gravity on the perimeter is similar to the on-ball gravity of a typical NBA player at these locations—but that Curry’s off-ball gravity near the hoop is actually slightly less than that of a typical player. S. Gilgeous-Alexander, in contrast, has a high gravity near the hoop, but his gravity at the perimeter is only slightly above that of a typical player. Finally, we see that L. Doncic is generally more balanced, having high gravity at most locations. We note that these results, which identify gravity through the statistical positioning of the defense, align extremely well with the widely recognized scoring patterns of each of these players^42,43^.

Finally, Fig. 4b shows the average local gravity across locations, weighted by the fraction of time the player spends at each of them. We find that on-ball (vertical axis) and off-ball (horizontal axis) gravity tend to be highly correlated, and that S. Curry—a key player for motivating the *qualitative* concept of gravity in basketball—truly has an exceptionally high gravity.

## Conclusions and future directions

Density-functional fluctuation theory (DFFT) quantifies the subtleties that underlie offensive and defensive strategies in NBA basketball by employing densities and systematic parameter reduction, requiring orders of magnitude fewer parameters than a direct probabilistic (DP) approach for the joint probability of offensive and defensive densities. Despite DFFT’s fewer parameters, we show that both DFFT and DP show similar fidelity at predicting player locations and relating a position to the probabilities of different scoring outcomes. After establishing the efficacy of DFFT and necessity of parameter reduction for player-specific results, we show how to employ DFFT to rank players by their positional contribution to their team’s defense at preventing either 2-point or 3-point scoring outcomes for the opposing team. We note that this quantification is based on the *secondary* effects of positional choices, capturing a player’s defensive contribution in comparison to reasonable locations for the player based on the other players and the ball. Additionally, we use DFFT to develop a density-based measure of player gravity, and demonstrate conclusively that, indeed, certain offensive players, especially the league’s leading scorers, have substantially higher “gravity” than typical offensive players. We found that each player’s gravity strongly depends on the player’s location on the court, in good agreement with known player scoring tendencies.

We note that there are multiple future directions that can directly extend this work. These can include improvements to the DFFT model of Eq. (2), such as accounting for the number of ways densities can occur in a bin. This ‘combinatorial counting’ is frequently found in other DFFT work^26,27,38^. There may also be interest in measuring players’ defensive ability orthogonally to their primary defensive role. This could be accomplished by considering within-group differences by primary role, or by taking into account the range of likely locations a player could occupy, rather than using the uniform ‘most probable 10% area’ of the half-court as we have done here.

**Fig. 5:**
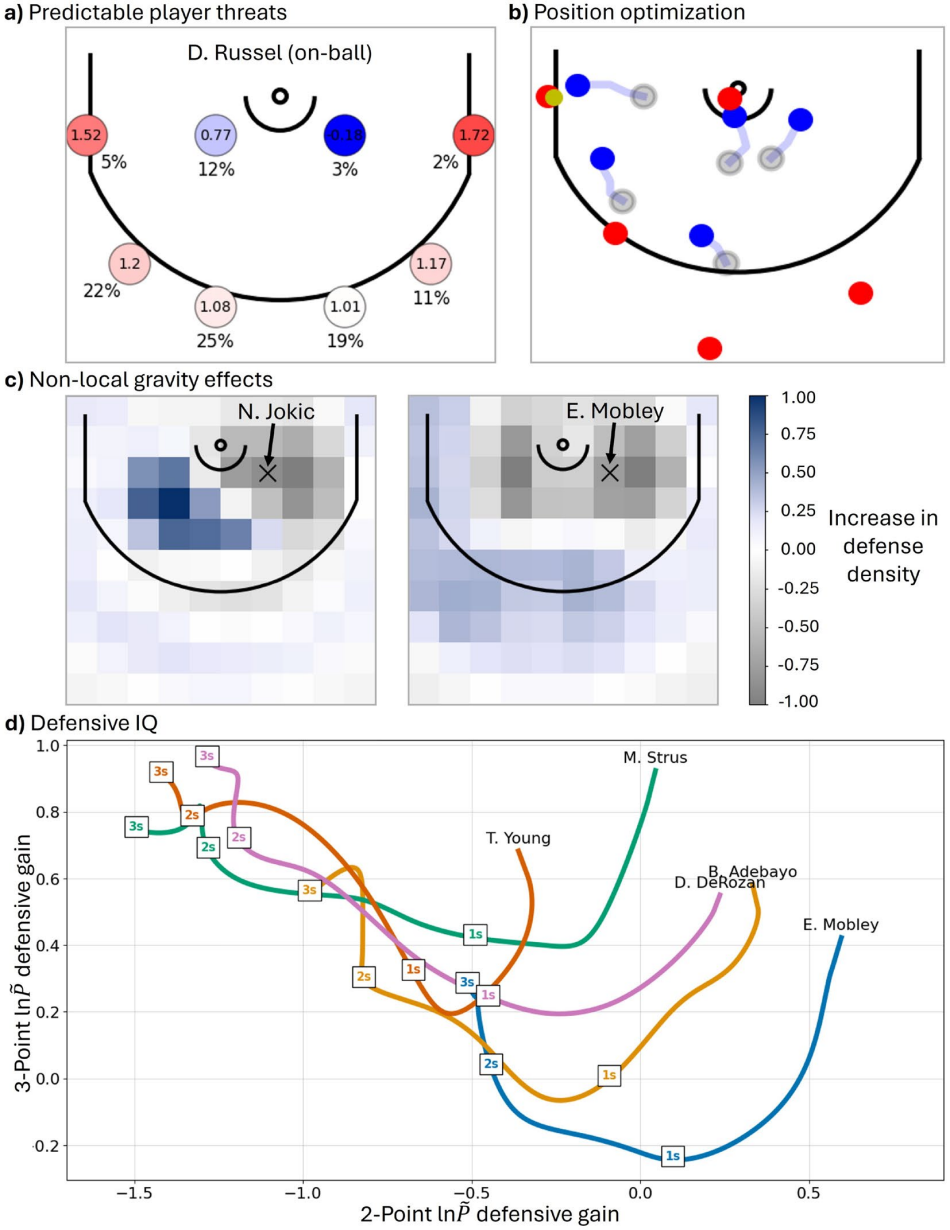
Additional applications of DFFT for basketball. (**a**) Predictable player threats: although most players have symmetric gravity, D. Russell not only has the ball more on the left side (e.g., 12% compared to 3% near the hoop) but is also viewed as a higher threat on the left side, as measured by his on-ball local gravity (0.77, compared to -0.18 on the right side). (**b**) Position optimization: rather than just individual players, DFFT can optimize the entire defensive position. Example shows repositioning from original defense (grey circles) to maximal ln$\tilde{P}$ difference 3-point defense (blue circles). (**c**) Non-local gravity effects: Jokic and Mobley have similar on-ball local-gravity at location [7.5, 37.5] but have distinct non-local gravity effects, with Jokic inducing a strong increase in defensive density near [-7.5, 32.5] which is likely associated with his distinct passing patterns. **(d)** Defensive IQ: same approach as Fig. 3 but now as a function of time leading up to the shot for selected players. Young, Strus, and DeRozan have a similarly strong 3-point defense and weak 2-point defense 2-3 seconds before a shot is attempted. When the play outcome was $R_{shot} = 2$, all three improved their defense positioning closer in time to the shot attempt (increase along horizontal axis), although Young improves less than the others. For $R_{shot} = 3$ (vertical axis), Mobley and Adebayo anticipate 3-point attempts earlier than the others—higher ‘defensive IQ’—with the last second prior to the shot attempt having a monotonic improvement in their 3-point defense, whereas players such as Young and DeRozan are still showing worsening 3-point defense $t=1$ second before successful 3-point shots from the opposing team.

Finally, there are many forward-looking directions for this work, a few of which are illustrated in Fig. 5. Figure 5a shows that analysis of our player-specific results can be used to make recommendations to improve the value of specific players to their team. In this example, we see that D. Russell not only spends more time on the left-side of the hoop, but he also garners substantially more gravity on the left-side when he has the ball; the defense knows this is where he is particularly a threat and could be caught off-guard if he were to play more on the right side—our recommendation based on these results. Figure 5b presents an application to position optimization, where the locations of the defense players is optimized for optimal defense against $R_{shot} = 3$. Figure 5c shows that some players may possess similar ‘local gravity’ but have different non-local effects on the defense. In particular, NBA Finals most valuable player (Finals MVP) N. Jokic is found to increase the defensive density not directly at his location but on the weak side when he has the ball, likely due to opposing players’ recognition of his tendency to pass the ball rather than attempt the shot himself. Lastly, Fig. 5d demonstrates how we can consider the defensive positioning of Fig. 3, but now as a function of time until the shot attempt. This approach could allow us to determine not only the defensive effectiveness of players, but also how quickly they adapt when the shot is turning into a 2-point or 3-point play, with some players showcasing a higher ‘defensive IQ’ as evidenced by their positioning correctly anticipating the play earlier than other players. Although these examples are natural extensions of the work we presented here, we leave detailed analyses of these demonstrative results for future research.

## Supplementary Information

Supplementary Information 1.

## Data Availability

Data of precise NBA player positions are proprietary to Second Spectrum. However, a subset of the processed data (following the transformation to densities) is available here: https://github.com/bb667/DFFT_Basketball.
