# Supplementary material for: Analyzing NBA player positions and interactions with density-functional fluctuation theory
Source: Sci Rep. 2025 Jun 5;15:19830. doi: 10.1038/s41598-025-04953-x (PMC12141578; doi:10.1038/s41598-025-04953-x)
Supplement: Supplementary file 1 — Supplementary Information 1. [file 41598_2025_4953_MOESM1_ESM.pdf]

Analyzing NBA Player Positions and Interactions with Density-Functional Fluctuation Theory

Supplementary Information (SI)

Boris Barron[1,2,*], Nathan Sitaraman[1], Tomás Arias[1]

[1]Cornell University, Department of Physics, Ithaca, 14850, United States
[2]Max Planck Institute for Demographic Research, Rostock, 18057, Germany
*bb667@cornell.edu

S1 Derivation of DFFT model

There are multiple ways to arrive at the model of equation 2 in the main text. One approach is to use an information theory perspective [38], another is to use a physics-based perspective [26]. Here, we will summarize the information theory approach.

Suppose we want to predict the density distribution in bin b' given that we know the bin's average offensive and defensive densities, $\langle n_o \rangle_{b'} = \sum_{n_o,n_d} n_o P_{b'}(n_o,n_d)$ and $\langle n_d \rangle_{b'} = \sum_{n_o,n_d} n_d P_{b'}(n_o,n_d)$, and further know the distribution in a different bin (bin b), $P_b(n_o,n_d)$. In particular, our goal here is to combine different pieces of information—an initial distribution and new average density values—to better approximate the distribution in bin b' than simply using the known, bin b, distribution. To construct our prediction for bin b', $\tilde{P}_{b'}(n_o,n_d)$, in a maximally unbiased way from an information theory perspective, we can minimize the Kullback–Leibler (KL) divergence the distribution we know $P_b(n_o,n_d)$ and our predicted distribution *under the constraints* of the known average offensive and defensive densities in bin b',

$$\tilde{P}_{b'}(n_o,n_d) = \underset{\rho(n_o,n_d)}{\operatorname{argmin}} \sum_{n_o,n_d} \rho(n_o,n_d) \ln\left(\frac{\rho(n_o,n_d)}{P_b(n_o,n_d)}\right), \tag{S1}$$

where the argmin of $\rho(n_o,n_d)$ represents a search through all distributions satisfying the target average densities and identifying the one that minimizes $\sum_{n_o,n_d} \rho(n_o,n_d) \ln\left(\frac{\rho(n_o,n_d)}{P_b(n_o,n_d)}\right)$. Performing this minimization with Lagrange multipliers identifies this minimizing distribution to be of the form

$$\tilde{P}_{b'}(n_o,n_d) = \frac{1}{Z} P_b(n_o,n_d) e^{-n_o v_o - n_d v_d}, \tag{S2}$$

where Z is a normalization factor which ensures that the distribution sums to unity, while v_o and v_d are parameters that account for the average densities. The parameters, v_o and v_d, then need to be numerically optimized so that the resulting distribution satisfies the known average densities in bin b'.

In the information theory literature, minimizing the KL-divergence is sometimes referred to as minimum discrimination information or minimum cross-entropy. To provide an interpretation of this procedure, the KL-divergence itself is a common measure of 'statistical distance' of one distribution from another. Finding the distribution that minimizes the KL-divergence under a set of constraints is equivalent to identifying the conditional distribution $P_b(n_o,n_d \,|\, \langle n_o \rangle_{b'}, \langle n_d \rangle_{b'})$—the unique distribution that satisfies the target average values and is constructed from sampling of $P_b(n_o,n_d)$ as the number of samples approaches infinity. Furthermore, once the values of the parameters Z, v_o, v_d are optimized, the obtained $\tilde{P}_{b'}(n_o,n_d)$ is guaranteed to be closer than $P_b(n_o,n_d)$ to the actual distribution in bin b', $P_{b'}(n_o,n_d)$, in the sense that

$$\sum_{n_o,n_d} P_{b'}(n_o,n_d) \ln\left(\frac{P_{b'}(n_o,n_d)}{\tilde{P}_{b'}(n_o,n_d)}\right) \leq \sum_{n_o,n_d} P_{b'}(n_o,n_d) \ln\left(\frac{P_{b'}(n_o,n_d)}{P_b(n_o,n_d)}\right), \tag{S3}$$

with equality holding only when bin b has the same average densities as bin b'.

To generalize the above methodology to multiple bins, we can replace our initial 'guess' distribution, which was based on the distribution of a single bin $P_b(n_o,n_d)$, by an optimal non-parametric function $e^{-f(n_o,n_d)}$. In other words, we will *also* optimize over the best starting distribution based on data observed in all bins. The resulting model for bin b' is then given by

$$\tilde{P}_{b'}(n_o,n_d) = \frac{1}{Z} e^{-n_o v_o - n_d v_d - f(n_o,n_d)}, \tag{S4}$$

where the values for the parameters are obtained from

$$\{f(n_o,n_d), v_{o,b}, v_{d,b}\} = \underset{f(n_o,n_d)}{\operatorname{argmin}} \sum_b \underset{v_{o,b},v_{d,b}}{\operatorname{argmin}} \sum_{n_o,n_d} P_b(n_o,n_d) \ln\left(\frac{P_b(n_o,n_d)}{\tilde{P}_b(n_o,n_d)}\right), \tag{S5}$$

which is equivalent to equation 3 in the main text. Note that this result corresponds to a maximum likelihood estimation (MLE) of the parameters. Conceptually, $f(n_o, n_d)$ encompasses the best initial estimate for the distribution of a bin, based on the distributions observed across all bins, while $v_{o,b}$ and $v_{d,b}$ further improve this estimate by capturing bin-specific average densities, with lower values of $v_{o/d,b}$ representing bins b which are relatively more "attractive" or likely locations for the offense/defense, respectively. The power of this DFFT approach is the construction of the model itself, equation S4, which takes as an input precisely the desired quantities (in our case, a starting guess distribution and known average densities) in a manner which is guaranteed to improve upon any initial guess distribution.

In summary, although DFFT can be applied more generally—beyond the salient bin-specific property being the average density—the DFFT form of equation S4 is among the simplest and most conventional. This model can be interpreted as capturing location-based preferences using v, which are typically referred to as 'vexations' in DFFT literature, while the bin-independent 'player-to-player interactions' are captured by the non-parametric function f, which is typically referred to as the frustration. This terminology stems from the fact that high values of these quantities correspond statistically to situations which are avoided, with v analogous to an external potential energy and f analogous to an internal free energy in physics. Crucially, DFFT provides a systematic approach to reduce the number of parameters needed to describe density distributions, with the parameterization defined here reducing the number of parameters from $N_{bins}N^2_{densities} = 40,000$ down to $2N_{bins} + N^2_{densities} = 600$.

S2 Considered Ball Locations

In this work, we take into account ball location in the training set positions by weighting the data by distance of the ball to the target ball location using a Gaussian with $\sigma = 2$ ft. To construct our grid of target ball locations throughout the half-court, while taking into account the frequency with which the ball is present at different locations, the half-court was initially parsed into a 100 by 100 grid. The ball locations that ended up being included were determined by selecting the most frequent locations from the grid (with a symmetric location included on the other side of the lengthwise central line) iteratively, while excluding grid locations that were closer than 5 feet to previously selected ball locations. This resulted in 89 locations overall, with 36 locations inside the 3-point line and 53 locations outside the three point line, as shown in Figure S1. Although training of the models included positions Gaussian-weighted by ball distance to one of these 89 ball locations, our efficacy and results sections (those using a 'testing set') only included positions for which the ball was within 2 feet of one of these 89 locations, for which the corresponding trained model was then used.

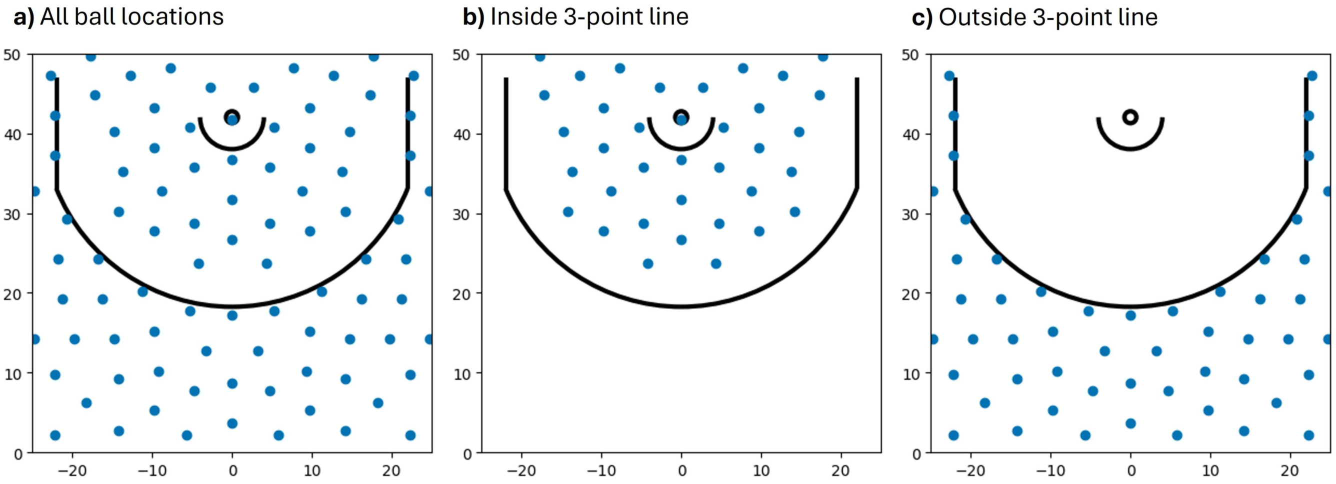

Figure S1. Grid of considered ball locations. 89 locations overall, 36 inside the 3-point line and 53 outside.

S3 Success Rates in Player Localization

As referenced in the main text, both the direct probabilistic (DP) and density-functional fluctuation theory (DFFT) predict "hidden" player locations quite well, with the DP approach performing marginally better. Figure S2 shows our detailed results. These results show, for example, that depending somewhat on play outcome, the DP approach typically localizes the player to within 1% of the half-court ≈30% of the time, as compared to DFFT at ≈25% of the time. We also find that, in half of all cases (median line), the DP approach correctly predicts the player location to within 2.1-2.9% of the half-court, depending on play outcome, with DFFT being able to localize in half of cases to within 2.5-3.1%.

S4 Overfitting in DP versus DFFT model

As the direct probabilistic approach requires a large number of parameters, overfitting is a concern. To illustrate this effect, Figure S3 shows the resulting artifacts in predicting a player's location. This occurs because of the uncharacteristically high defensive and offensive densities near the hoop in this particular position, which is rarely observed in the dataset. Notably, while reproducing very similar likelihood maps, the DFFT approach exhibits far fewer artifacts because it does not require that the specific densities actually occur within the specific bins, a direct benefit of how DFFT reuses information across different bins.

S5 Additional Correlation examples

To further showcase that our analysis captures non-trivial aspects of overall team positioning, we also consider how the probability of a 2-point result changes with $\Delta \ln \tilde{P}$ when the ball is *outside* the 3-point line. It is possible to consider these situations because the datasets include data within 3 seconds of a shot being attempted, so that there is time for a player outside the 3-point line to run in and take the shot or to pass to another player who would then make a 2-point shot. The correlations for a 2-point shot when the ball is outside the 3-point line, and for a 3-point shot when the ball is inside the 3-point line, are shown in Figure S4. These correlations are unsurprisingly weaker than the correlation in the main text, with DFFT now showing $1.2\%/\Delta \ln \tilde{P}$ for 2-point results and $1.0\%/\Delta \ln \tilde{P}$ for 3-point results (compared to $2.3\%/\Delta \ln \tilde{P}$ and $1.2\%/\Delta \ln \tilde{P}$ in the main text, respectively). (Both DP and DFFT show similar performance.) Figure S5a further demonstrates correlations that arise when the ball is at specific locations outside the 3-point line, and Figure S5b demonstrates aggregations of ball locations based on average scoring probabilities from these locations.

Finally, Figure S6 returns to consideration of correlation between our model and actual outcomes when the ball does not need to cross the 3-point line before the shot, demonstrating correlations for positions *at the time of the shot*. Interestingly, the 2-point correlation indeed increases ($3.0\%/\Delta \ln \tilde{P}$ vs $2.3\%/\Delta \ln \tilde{P}$ in the main text), but the 3-point correlation actually becomes weaker ($0.8\%/\Delta \ln \tilde{P}$ vs $1.2\%/\Delta \ln \tilde{P}$ of the main text). This suggests that, as expected for 2-point shots, the position at the time of the shot is most important, but that for 3-point shots, the position leading up to it, rather than the position at the time of the shot, may actually be more meaningful.

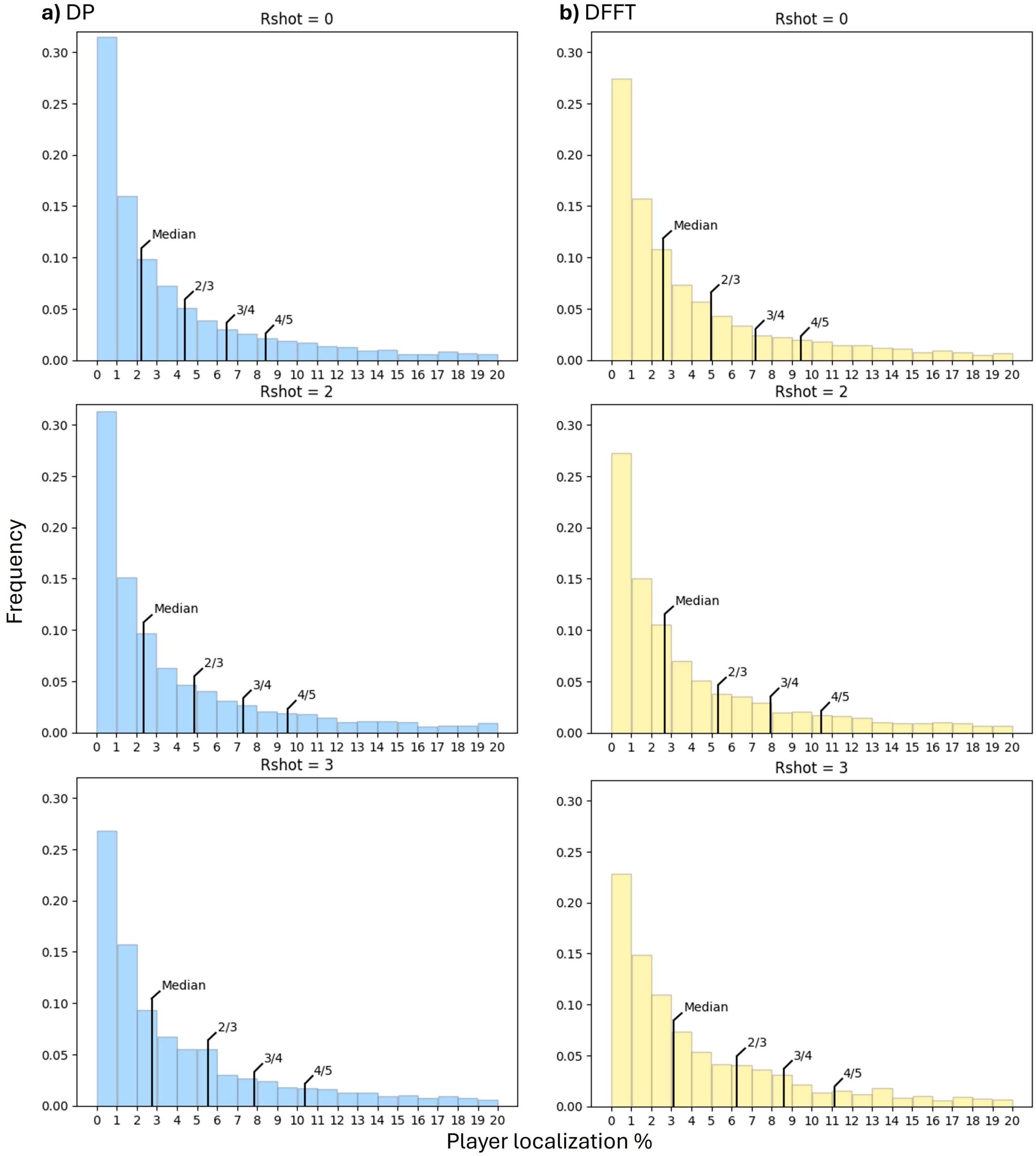

Figure S2. Histogram of 'hidden' defensive player localization accuracy, where the location of the player is inferred from the location of the 9 other players and the ball. Columns show results from **(a)** direct probabilistic (DP) and **(b)** DFFT approaches, for shot outcomes of 0, 2, and 3 points (top, middle, and bottom, rows respectively). Frequency of prediction success (vertical axis) versus percentage of court ranked more probable (horizontal), with median, second tercile, third quartile, and fourth quintile of frequency indicated (dark vertical lines). The DP model marginally outperforms the DFFT model, typically localizing the player to within 1% of the half-court ≈30% of the time, as compared to DFFT at ≈25% of the time. In almost all cases, the median line is at less than 3%, demonstrating that the location of the player is predicted to within 3% of the half-court more than half the time. For reference, the colormap of Figure 2 in the main text represents 5% of the half-court. Note that the prediction success of player localization is evaluated on the testing set (20% of positions corresponding to each dataset), representing data not used in training the model.

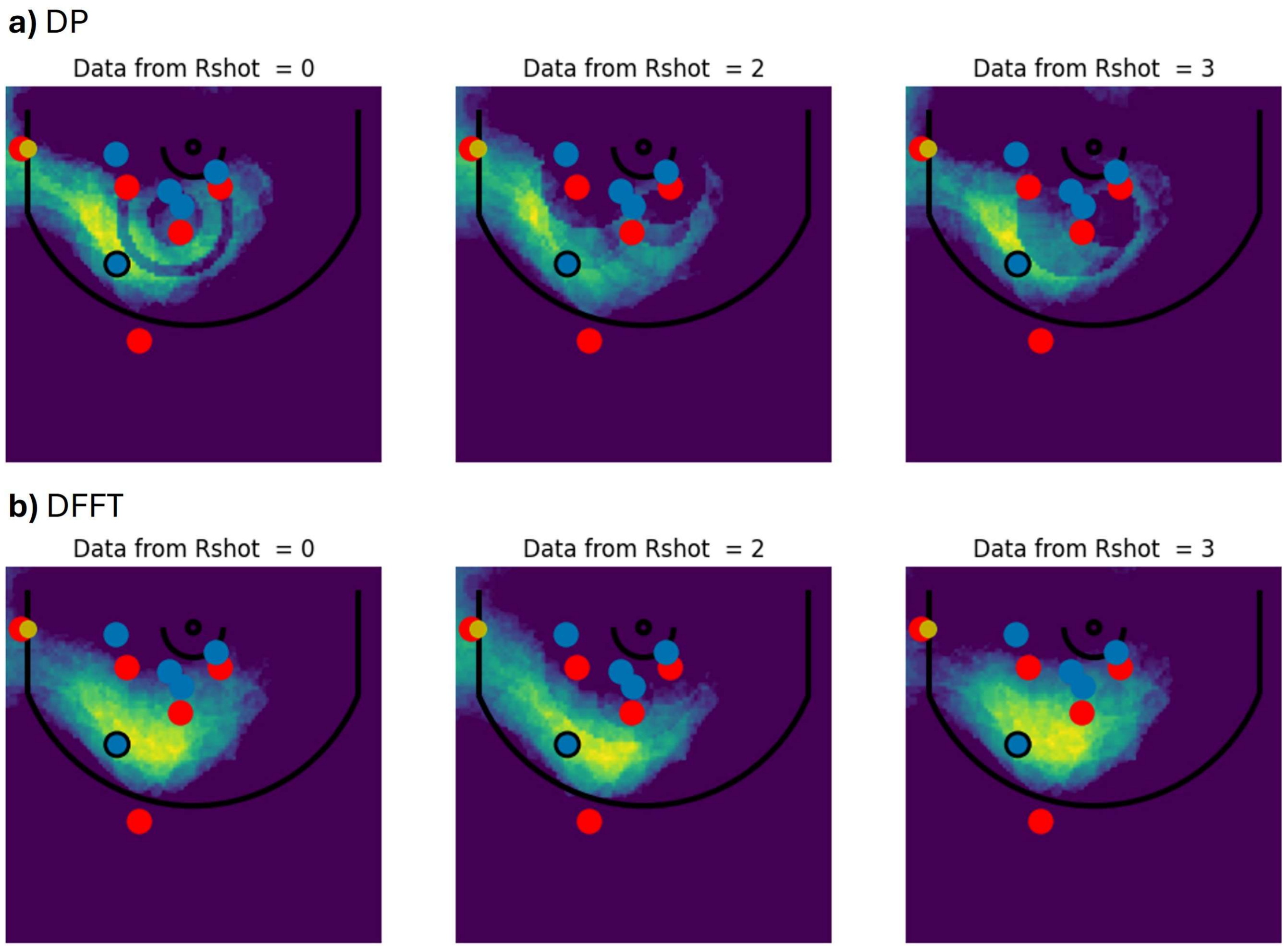

Figure S3. Artifacts in predicting player location (blue circle with black outline) that arise due to overfitting, with **(a)** showing DP and **(b)** showing DFFT predictions. In each case the colormap is displayed for the most probable 20% of locations in the half-court. The probabilistic approach has artifacts that are highly dependent on the specific dataset used, demonstrating overfitting, which is an issue that DFFT is much less susceptible to due to the reduction in parameters.

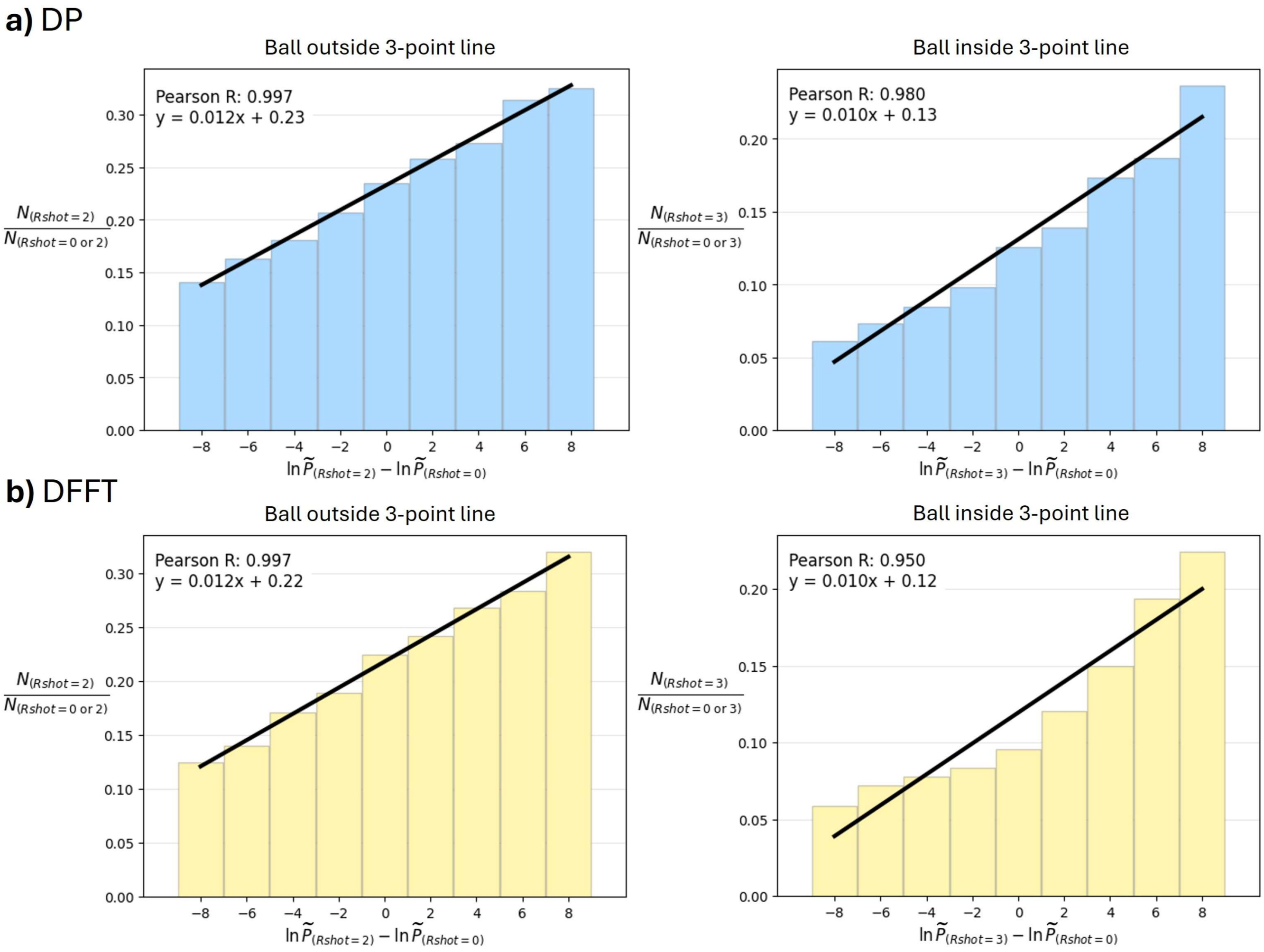

Figure S4. Correlation between $\ln\tilde{P}$ difference and 2-point outcome (left) and 3-point outcome (right) for *opposite* ball position, meaning the ball needs to cross the 3-point line for a scoring outcome, using the **(a)** DP and **(b)** DFFT approaches. (Same conventions as main text Figures 2c and 2d.)

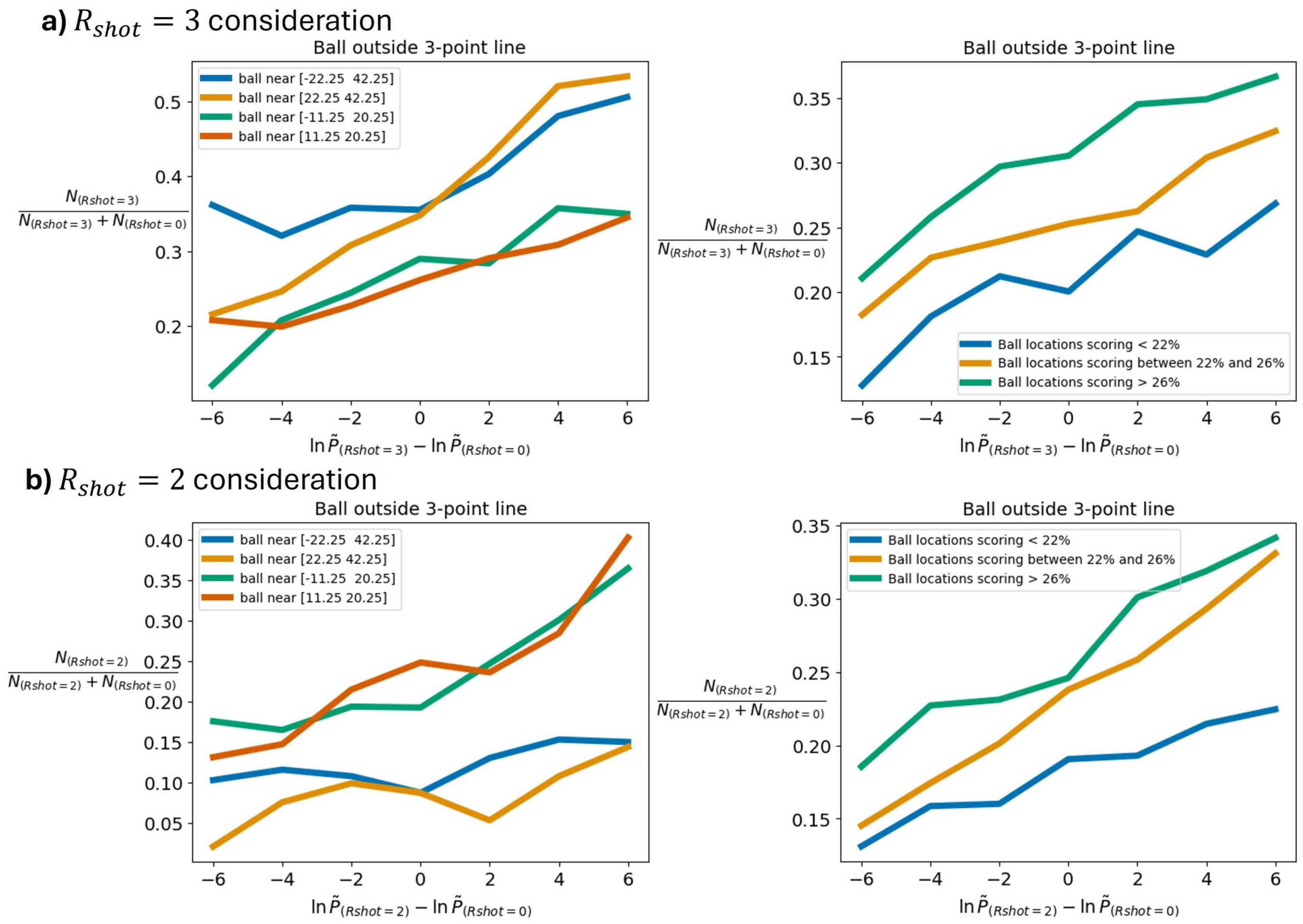

Figure S5. Correlation between scoring outcome and $\ln \tilde{P}$ difference at *specific ball locations* outside the 3-point line for **(a)** 3-point and **(b)** 2-point outcomes using DFFT. There is correlation between $\ln \tilde{P}$ differences and scoring probability even when considering specific ball locations (left). If locations are grouped by average scoring probability (right) there is less noise, with a strong positive correlation in all cases.

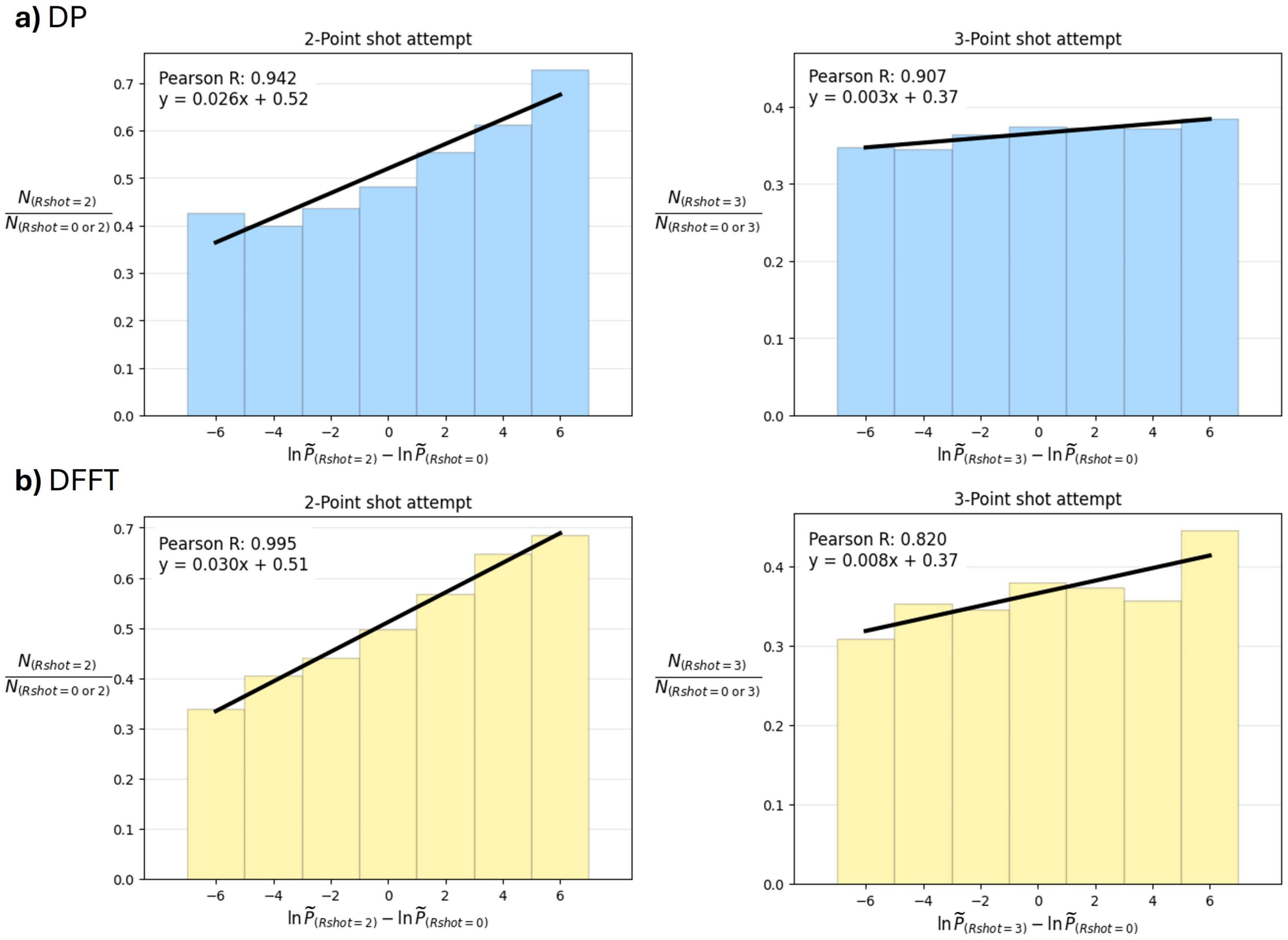

Figure S6. Correlation between $\ln \tilde{P}$ difference and play outcome *at the time of shot*: **(a)** shows probabilistic modeling for 2-pointers (left) and 3-pointers (right), and **(b)** shows the same for DFFT modeling. (Results in main text Figure 2(c,d) are for all times from 0-3 second before the shot.)

References

1. Kovalchik, S. A. Player tracking data in sports. *Annu. Rev. Stat. Its Appl.* **10**, 677–697 (2023).
2. Kambhamettu, A. R., Shrivastava, A. & Gwilliam, M. Quantifying nba shot quality: A deep network approach. In *Proceedings of the 7th ACM International Workshop on Multimedia Content Analysis in Sports*, 91–95 (2024).
3. Keshri, S. K. *Essays in Basketball Analytics*. Ph.D. thesis, Columbia University (2019).
4. Gudmundsson, J. & Horton, M. Spatio-temporal analysis of team sports. *ACM Comput. Surv.* **50**, 1–34 (2017).
5. Sá-Freire, B. M., Barbosa, G. R., Gonçalves, J. L. L., Schuster, J. & Rios-Neto, H. Grapheiv: A framework for estimating the expected immediate value in basketball using graph neural networks. In *International Workshop on Machine Learning and Data Mining for Sports Analytics*, 29–40 (Springer, 2024).
6. Yue, Y., Lucey, P., Carr, P., Bialkowski, A. & Matthews, I. Learning fine-grained spatial models for dynamic sports play prediction. In *2014 IEEE international conference on data mining*, 670–679 (IEEE, 2014).
7. Sampaio, J. *et al.* Exploring game performance in the national basketball association using player tracking data. *PloS ONE* **10**, e0132894 (2015).
8. Cervone, D., D'Amour, A., Bornn, L. & Goldsberry, K. A multiresolution stochastic process model for predicting basketball possession outcomes. *J. Am. Stat. Assoc.* **111**, 585–599 (2016).
9. Spearman, W. Beyond expected goals. In *Proceedings of the 12th MIT Sloan Sports Analytics Conference*, 1–17 (2018).
10. Cervone, D., Bornn, L. & Goldsberry, K. Nba court realty. In *10th MIT Sloan Sports Analytics Conference* (2016).
11. Bialek, W. *et al.* Statistical mechanics for natural flocks of birds. *Proc. Natl. Acad. Sci.* **109**, 4786–4791 (2012).
12. Frishman, A. & Ronceray, P. Learning force fields from stochastic trajectories. *Phys. Rev. X* **10**, 021009 (2020).
13. Eagle, N., Pentland, A. & Lazer, D. Inferring friendship network structure by using mobile phone data. *Proc. Natl. Acad. Sci.* **106**, 15274–15278 (2009).
14. Psorakis, I., Roberts, S. J., Rezek, I. & Sheldon, B. C. Inferring social network structure in ecological systems from spatio-temporal data streams. *J. Royal Soc. Interface* **9**, 3055–3066 (2012).
15. Jones, R. O. Density functional theory: Its origins, rise to prominence, and future. *Rev. Mod. Phys.* **87**, 897 (2015).
16. Hohenberg, P. & Kohn, W. Inhomogeneous electron gas. *Phys. Rev.* **136**, B864 (1964).
17. Kohn, W. & Sham, L. J. Self-consistent equations including exchange and correlation effects. *Phys. Rev.* **140**, A1133–A1138, DOI: 10.1103/PhysRev.140.A1133 (1965).
18. Runge, E. & Gross, E. K. Density-functional theory for time-dependent systems. *Phys. Rev. Lett.* **52**, 997 (1984).
19. Vignale, G. & Rasolt, M. Density-functional theory in strong magnetic fields. *Phys. Rev. Lett.* **59**, 2360 (1987).
20. Evans, R. The nature of the liquid-vapour interface and other topics in the statistical mechanics of non-uniform, classical fluids. *Adv. Phys.* **28**, 143–200, DOI: 10.1080/00018737900101365 (1979).
21. Rosenfeld, Y. Free-energy model for the inhomogeneous hard-sphere fluid mixture and density-functional theory of freezing. *Phys. Rev. Lett.* **63**, 980–983, DOI: 10.1103/PhysRevLett.63.980 (1989).
22. Evans, R. Density functionals in the theory of nonuniform fluids. *Fundamentals inhomogeneous fluids* **1**, 85–176 (1992).
23. Löwen, H. Applications of density functional theory in soft condensed matter. In *Understanding Soft Condensed Matter Via Modeling And Computation*, 9–45 (World Scientific, 2011).
24. Evans, R., Oettel, M., Roth, R. & Kahl, G. New developments in classical density functional theory. *J. Physics: Condens. Matter* **28**, 240401, DOI: 10.1088/0953-8984/28/24/240401 (2016).
25. te Vrugt, M., Löwen, H. & Wittkowski, R. Classical dynamical density functional theory: from fundamentals to applications. *Adv. Phys.* **69**, 121–247 (2020).

26. Méndez-Valderrama, J. F., Kinkhabwala, Y. A., Silver, J., Cohen, I. & Arias, T. Density-functional fluctuation theory of crowds. *Nat. Commun.* **9**, 1–10 (2018).

27. Chen, Y. *et al.* Small-area population forecasting in a segregated city using density-functional fluctuation theory. *J. Comput. Soc. Sci.* **7**, 2255–2275 (2024).

28. Kinkhabwala, Y. A., Barron, B., Hall, M., Arias, T. A. & Cohen, I. Forecasting racial dynamics at the neighborhood scale using density-functional fluctuation theory. *arXiv preprint arXiv:2108.04084* (2021).

29. Da Costa, L. & Rajotte, J.-F. Crowd prediction under uncertainty. In *Advances in Artificial Intelligence: 32nd Canadian Conference on Artificial Intelligence, Canadian AI 2019, Kingston, ON, Canada, May 28–31, 2019, Proceedings 32*, 308–319 (Springer, 2019).

30. Geertsen, I. Defensive deterrence i: Quantifying defenders' off-ball impact at the rim and beyond. Bruin Sports Analytics 2021.

31. Franks, A., Miller, A., Bornn, L. & Goldsberry, K. Characterizing the spatial structure of defensive skill in professional basketball. *Annals Appl. Stat.* **9**, 94–121, DOI: 10.1214/14-AOAS799 (2015).

32. Cheema, A. Quantifying gravity in the nba with ridge regression (2019). The spax 2019.

33. Basketball For Coaches. How to develop a high basketball iq (and 14 examples). Basketball For Coaches 2019.

34. Carlin, M. & Ayubi, C. Rethinking defensive impact. Sports Info Solutions 2021.

35. Basketball Reference. Glossary (2025). Basketball Reference, 2025.

36. Kubatko, J., Oliver, D., Pelton, K. & Rosenbaum, D. T. A starting point for analyzing basketball statistics. *J. Quant. Analysis Sports* **3**, 1–24, DOI: 10.2202/1559-0410.1070 (2007).

37. Daly-Grafstein, D. & Bornn, L. Using in-game shot trajectories to better understand defensive impact in the nba. *J. Sports Anal.* **6**, 235–242, DOI: 10.3233/JSA-200400 (2020).

38. Barron, B. *et al.* Extending the use of information theory in segregation analyses to construct comprehensive models of segregation. *arXiv preprint arXiv:2212.06980* (2022).

39. Pina, M. Jamal murray isn't the new steph curry, but he might be close. FiveThirtyEight 2020.

40. Haberstroh, T. Steph curry leads 2014–15 respect rating ranks in the nba. ESPN.com (Insider) 2014.

41. ESPN.com. Nba players (2025). Accessed: 2025-05-23.

42. Nba player stat leaders, 2022–23 regular season. ESPN 2023.

43. Basketball Index. 2024–25 player rankings. BBALL INDEX 2024.
